# Variable- versus constant-frequency deep-brain stimulation in patients with advanced Parkinson’s disease: study protocol for a randomized controlled trial

**DOI:** 10.1186/s13063-019-3884-4

**Published:** 2019-12-19

**Authors:** Fumin Jia, Jianguo Zhang, Huimin Wang, Zhanhua Liang, Weiguo Liu, Xuelian Wang, Yiming Liu, Yi Guo, Zhipei Ling, Xiaodong Cai, Xi Wu, Jianjun Wu, Wen Lv, Xin Xu, Wenbin Zhang, Luming Li

**Affiliations:** 1 National Engineering laboratory for Neuromodulation, Tsinghua University, Beijing, China; 2 Department of Functional Neurosurgery, Beijing Tiantan Hospital, Beijing, China; 3 Department of Neurosurgery, The First Affiliated Hospital of Dalian Medical University, Dalian, Liaoning China; 4 Department of Neurosurgery, Nanjing Brain Hospital, Nanjing, Jiangsu China; 5 Department of Neurosurgery, Tangdu Hospital, Fourth Military Medical University of Chinese PLA, Xi’an, Shanxi China; 6 Department of Neurosurgery, Qilu Hospital, Shandong University, Jinan, Shandong China; 7 Department of Neurosurgery, Peking Union Medical College Hospital, Beijing, China; 8 Department of Neurosurgery, Chinese PLA General Hospital, Beijing, China; 9 Department of Neurosurgery, the Second People’s Hospital of Shenzhen, Guangzhou, China; 10 Department of Neurosurgery, Changhai Hospital of Shanghai, Shanghai, China; 11 Department of Neurosurgery, Huashan Hospital, Fudan University, Shanghai, China; 12 Department of Neurology, Sir Run Run Shaw Hospital, Affiliated with School of Medicine, Zhejiang University, Hangzhou, Zhejiang China; 13 Tsinghua-Berkeley Shenzhen Institute, Precision Medicine & Healthcare Research Center, Shenzhen, Guangdong China; 14 Tsinghua university, School of Aerospace Engineering, Man-Machine-Environment Engineering Institute, Beijing, China; 15 Beijing Institute for Brain Disorders, Center of Epilepsy, Beijing, China

**Keywords:** Parkinson’s disease, Deep-brain stimulation, Variable frequency stimulation, Subthalamic nucleus

## Abstract

**Background:**

Deep-brain stimulation targeting the subthalamic nucleus (STN) can be used to treat motor symptoms and dyskinesia in the advanced stages of Parkinson’s disease (PD). High-frequency stimulation (HFS) of the STN can lead to consistent, long-term improvement of PD symptoms. However, the effects of HFS on the axial symptoms of PD, specifically freezing of gait, can be limited or cause further impairment. While this can be alleviated via relatively low-frequency stimulation (LFS) in selected patients, LFS does not control all motor symptoms of PD. Recently, the National Engineering Laboratory for Neuromodulation reported preliminary findings regarding an efficient way to combine the advantages of HFS and LFS to form variable-frequency stimulation (VFS). However, this novel therapeutic strategy has not been formally tested in a randomized trial.

**Methods/design:**

We propose a multicenter, double-blind clinical trial involving 11 study hospitals and an established deep-brain stimulation team. The participants will be divided into a VFS and a constant-frequency stimulation group. The primary outcome will be changes in stand–walk–sit task scores after 3 months of treatment in the “medication off” condition. Secondary outcome measures include specific item scores on the Freezing of Gait Questionnaire and quality of life. The aim of this trial is to investigate the efficacy and safety of VFS compared with constant-frequency stimulation.

**Discussion:**

This is the first randomized controlled trial to comprehensively evaluate the effectiveness and safety of VFS of the STN in patients with advanced PD. VFS may represent a new option for clinical treatment of PD in the future.

**Trial registration:**

ClinicalTrials.gov, NCT03053726. Registered on February 15, 2017.

## Background

Deep-brain stimulation (DBS) of the subthalamic nucleus (STN) is an established treatment for Parkinson’s disease (PD). However, debilitating axial symptoms such as gait impairment, postural instability, postural abnormalities, dysphagia, and dysarthria are frequently observed in individuals with advanced PD [1]. Axial motor impairments can be highly debilitating and are a common cause of disability in patients with PD. Freezing of gait (FOG) is a unique and disabling clinical phenomenon that typically occurs when initiating gait or when turning while walking. FOG is characterized by brief episodes in which the patient demonstrates an inability to step, or makes repeated use of extremely short steps [2]. The inability to start walking is a prominent feature in PD patients with FOG. However, once this so-called ‘freezing’ pattern is interrupted, patients often regain the ability to walk normally [3]. FOG has also been observed in patients with progressive supranuclear ophthalmoplegia, multiple system atrophy, corticobasal degeneration, and numerous other diseases [4]. Additional axial signs include postural instability and changes in postural alignment, such as camptocormia or Pisa syndrome. Dysphagia and speech disorders, especially dysarthria and stuttering, are equally disabling axial motor features. However, conventional DBS stimulation has limited therapeutic potential for treating these symptoms. Thus, these symptoms represent a public health issue for which a specific treatment is currently lacking.

High-frequency DBS of the STN (STN-HFS) mainly improves levodopa-sensitive PD symptoms. However, it is typically less effective in improving axial symptoms, such as FOG [5]. Previous studies have shown that low-frequency subthalamic stimulation (STN-LFS) improves axial motor activity in some PD patients, but with short-term therapeutic efficacy. Thereafter, patients may present with increased tremor, rigidity, and bradykinesia, and the intensity of symptoms often outweighs the initial benefits of LFS therapy for FOG [6, 7]. The conventional HFS or LFS programming is called constant-frequency stimulation (CFS) due to the fixed stimulation frequency and is the current standard method of DBS. Previously, we successfully used variable-frequency stimulation (VFS) of the STN to treat FOG in patients with PD [8]. Furthermore, we recently conducted a study with 28 participants (under review) that revealed that, compared with HFS and LFS, VFS DBS improved FOG and appendicular motor symptoms in patients with PD, with sustained benefits for 12 months. Our pilot study suggested that VFS composed of both HFS and LFS was safe, and we did not observe any clinically relevant neuropsychiatric adverse effects. To the best of our knowledge, no controlled prospective studies have compared VFS with CFS in patients with PD. To this end, we designed a prospective, controlled study investigating the outcome of VFS in patients with advanced PD.

### Aims

The motives for this clinical study are twofold. First, we plan to evaluate the short-term effects of VFS and CFS on motor and axial symptoms in patients with advanced PD. Second, we hope to gain insight regarding whether VFS is more effective than CFS after 3 months of treatment, with a 6-month follow-up period. The study has a randomized, double-blind design.

## Design and methods

### Trial design

**Fig. 1:**
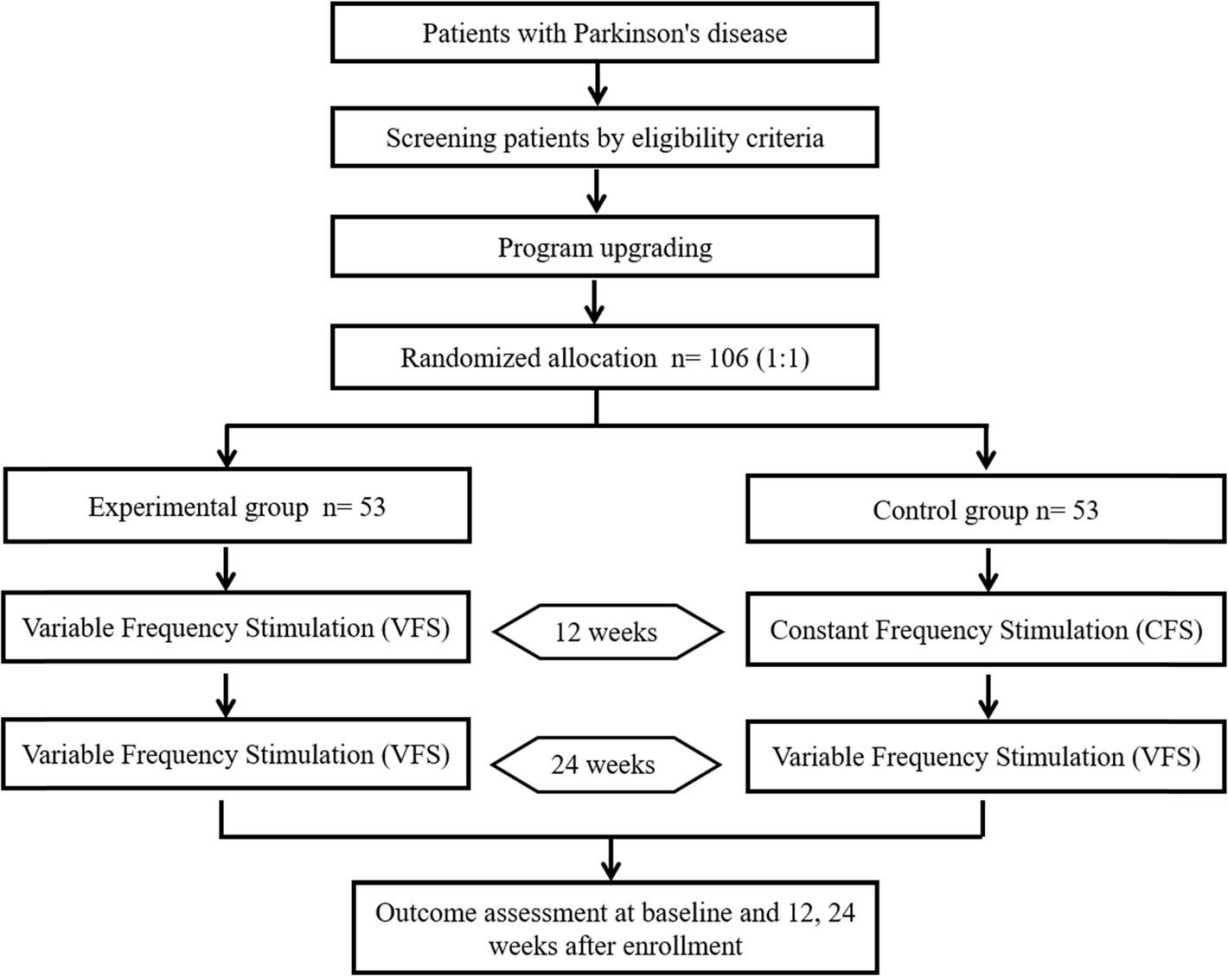
Flow chart of the RESTEP trial

The RESTEP study (ClinicalTrials.gov, NCT03053726) is a double-blind, randomized, multicenter trial designed to evaluate the safety and effectiveness of STN-VFS treatment in participants with advanced PD. According to the trial flow chart (Fig. 1), participants with advanced PD who had previously undergone DBS will be screened based on strict inclusion and exclusion criteria. A total of five follow-up surveys will be scheduled as shown in Table 1. After recruitment, pulse generators previously implanted in all participants will be upgraded from those with a single mode (CFS only) to those that are capable of dual-mode stimulation (CFS or VFS). Following this upgrade, participants will be randomly and equally divided into two groups: the CFS group and the VFS group. After 3 months of stimulation under the blinded conditions, all participants will receive VFS and follow-up assessments for 6 months. The protocol design is based on the guidelines of the Consolidated Standards of Reporting Trials and Standard Protocol Items: Recommendations for Interventional Trials (SPIRIT) (see Additional file 1), and the study results will also be reported according to these guidelines. Informed consent will be obtained from all participants in accordance with the policies of the board.

**Table 1:** Detailed schedule of the RESTEP trial

Schedule	Baseline		Visit		
	Screening	Upgrading	EG for VFS, CG for CFS	EG for VFS, CG for VFS	EG for VFS, CG for VFS
Time	3 ± 2 days	5 ± 2 days	90 ± 14 days	90 ± 14 days	180 ± 14 days
Follow-up	V1	V2	V3	V4	V5
Informed consent	X				
Criteria for inclusion	X				
Criteria for exclusion	X				
MMSE	X		X		X
Hoehn-Yahr staging	X				
Medical history/demographic data	X				
Physical examination	X				
Records of side effects	X	X	X		X
PDQ-39	X		X		X
FOG-Q	X		X		X
GAFQ	X		X		X
Medication	X		X		X
UPDRS (part I/II/IV)	X		X		X
Upgrade to dual mode		X			
Random grouping		X			
Programming	X	X	X	X	X
SWS test	X	X	X	X	X
UPDRS (part III)	X	X	X	X	X
Admission confirmed	X				
AE		X	X	X	X
SAE		X	X	X	X

*AE* adverse event, *CFS* constant-frequency stimulation, *CG* control group, *EG* experimental group, *FOG-Q* Freezing of Gait Questionnaire, *GAFQ* Gait and Falls Questionnaire, *MMSE* Mini-Mental State Examination, *PDQ-39* Parkinson’s Disease Questionnaire-39, *SAE* serious adverse event, *SWS* stand–walk–sit, *UPDRS* Unified Parkinson’s Disease Rating Scale, *VFS* variable-frequency stimulation

### Trial population

The trial will be conducted in approximately 11 centers. These include Beijing Tiantan Hospital, the First Affiliated Hospital of Dalian Medical University, Nanjing Brain Hospital, Tangdu Hospital (Fourth Military Medical University of Chinese PLA), Qilu Hospital, Peking Union Medical College Hospital, Chinese PLA General Hospital, the Second People’s Hospital of Shenzhen, Changhai Hospital of Shanghai, Huashan Hospital, and Sir Run Shaw Hospital (affiliated with the School of Medicine, Zhejiang University). Other hospitals will be invited to join the study according to interest, feasibility, and resources. The clinical investigators from each center will be responsible for screening eligible participants.

### Patient population and recruitment

We intend to enroll a total of 106 participants aged over 18 years. The prospective cohort will comprise patients with PD who have received STN-DBS treatment and have implanted impulse generators that are compatible with VFS (PINS Medical).

#### Inclusion criteria

The inclusion criteria are as follows:

1. Patients with idiopathic PD
2. Aged ≥18 years of either sex
3. Patients who have already undergone DBS and currently have an implanted STN-DBS stimulator
4. A Mini-Mental State Examination score ≥24
5. A Hoehn-Yahr score ≥2.0 when undergoing CFS in a “medication off” state
6. A score ≥1 on the 14-item Unified Parkinson’s Disease Rating Scale II (UPDRS-II)
7. A score ≥2 on the 15-item UPDRS-II
8. The ability to walk ≥10 m independently when receiving CFS in a “medication off” state

#### Exclusion criteria

The exclusion criteria are as follows:

1. Pregnant women, breastfeeding mothers, or woman who are unable to take effective measures to prevent pregnancy
2. Presence of other diseases that can affect walking distance, such as joint disease in the lower body, spinal disease, neuropathy, or serious heart or lung disease
3. Serious health conditions such as tumor, liver or kidney disease, and so forth
4. Epilepsy or other seizure disorders
5. Mental disorders or dementia
6. Inability to comprehend the experimental protocol or voluntarily provide informed consent
7. Lack of cooperation with follow-up requirements
8. Additional reasons for exclusion at the discretion of the clinical investigator

#### Recruitment of participants

Participants will be recruited by placing advertisements on social media and posters in clinics and directly at the participating centers. The recruitment information mainly includes eligibility criteria and contact details. A well-trained investigator in each participating center will be responsible for screening all potentially eligible patients based on the eligibility criteria and obtaining the informed consent.

### Interventions

#### Constant-frequency stimulation

To deliver CFS, electrical stimulation will be set to a constant frequency by the physician programming the stimulation frequency parameters (i.e., the pulse width and amplitude). The participants with receive STN-DBS with single-frequency, single pulse width, and single-amplitude stimulation.

#### Variable-frequency stimulation

The VFS parameters will be selected based on previous findings regarding the relationship between stimulation frequency and movement rhythm regulation in humans. The stimulation frequency will be set to alternate between high and low frequencies.

#### Concomitant interventions

Participants will be allowed to continue taking pretrial medications. Use of all drugs, if any, will be documented in the case report form (CRF).

### Randomization and blinding

Eligible participants will be randomly assigned to one of two groups (CFS group or VFS group) after completing the baseline measurements. A 1:1 assignment sequence (based on computer-generated random numbers) will be produced by The Peking University Clinical Research Institute. The computer-generated random numbers will be used to create the participant numbers and order lists, which will be placed in opaque sealed envelopes and sent to the research centers. The research clerk will keep copies of the order lists and participant numbers.

Throughout the study, with the exception of the study programmer, all participants and study staff (including investigators, trainers, and statisticians) will be blinded to the treatment allocation. Independent raters who have no therapeutic relationships with the participants and who are blind to the treatment conditions will conduct outcome assessments. The independent raters will be clinical neurologists who have received additional training on the use of the outcome assessments, had the opportunity to listen to conducted assessments, and received direct feedback regarding their assessments from supervisors. Furthermore, the Data and Safety Monitoring Board (DSMB) will review a random selection of 20% of the recorded assessments. Loss of blinding may occur due to the magnitude of the therapeutic effect or any other subjective perception that indicates that VFS or CFS is delivered.

### Trial outcomes

#### Primary clinical outcomes

The primary outcome will be stand–walk–sit (SWS) task scores at 3 months compared with the baseline scores. The domain of the SWS score is the gait. The study will compare the mean scores in each group between 3 months and baseline, using the mean scores from each group. The SWS test is a simple and quick functional mobility test that requires an individual to stand up, walk 5 m, turn, walk back, and sit down. The time taken to complete the test is strongly correlated with the level of functional mobility. This test will be videotaped and scored by two blinded neurologists.

#### Secondary clinical outcomes

As secondary outcomes, we will use the Freezing of Gait Questionnaire, Gait and Falls Questionnaire, and Parkinson’s Disease Quality of Life questionnaire, as well as total UPDRS and UPDRS-III scores to assess PD symptom severity at 3 and 6 months compared with the baseline. These questionnaires assess physiological symptoms and emotional function in patients with PD. Participants in both trial groups will be asked to complete these questionnaires at baseline and post-treatment, and the trial group will also be asked to complete the measures 3 months and 6 months post-treatment. The study will compare the mean change scores from baseline at each time point, using the mean scores from each group.

### Safety aspects

#### Adverse events

An adverse event (AE) could include an abnormal laboratory finding, symptom, or disease temporally associated with the administration of an investigational product, regardless of whether it is related to that investigational product. In the present study, an unexpected AE is anything that is not identified in nature, severity, or frequency in the Investigator’s Brochure. During routine assessments, the investigator will question the participants about the occurrence of AEs and record the information in the source documents and patient CRF.

#### Serious adverse events

A serious adverse event (SAE) is one which cause death, a life-threatening adverse experience, inpatient hospitalization or prolongation of existing hospitalization, a persistent or significant disability/incapacity, or a congenital anomaly/birth defect. Other important medical events may also be considered SAEs when, based on appropriate medical judgment, they jeopardize the participant or require intervention to prevent one of the outcomes listed.

When AEs occur throughout the clinical trial, investigators may take necessary measures according to the condition of the participant. Based on the severity of the AE, the investigators may choose hospitalization, outpatient treatment, home visits, communication, or other follow-up methods.

### Data and Safety Monitoring Board

The DSMB will review the safety, ethics, and outcomes of the study. It is independent from the sponsor and has no competing interests. DSMB members will monitor blinded assessment data for SAEs or potential harmful effects. A charter that will outline member responsibilities, procedures, and confidentiality will govern the DSMB. The DSMB will also review unblinded data at regular intervals during the follow-up period and will monitor neurological and functional differences between the two groups, as well as drop-out and event rates. Furthermore, the DSMB members will also manage the oversight of the trial progress, such as the status of recruitment and involvement of the sites.

### Data quality and management

Data collection will be restricted to the those who meet the eligibility criteria. Participants who withdraw from the study for any reason will have their data recorded in their medical records and be reviewed by the trial monitor.

#### Data collection and monitoring

CRFs must be completed according to the schedule. All SAEs that occur during the observation period must be reported to the medical expert overseeing the investigation (the medical director of Beijing PINS Medical Co., Ltd). The investigators are responsible for all information collected about participants enrolled in this study. The investigators and sponsor will conduct regular telephone or home follow-up of patients.

The monitoring system will assure the quality of the assessments. The data will be entered into a validated database. The Data Management Group will be responsible for data processing, in accordance with procedural documentation. The database will be locked once quality assurance procedures have been completed.

#### Withdrawal and termination from the study

In case of endangerment of personal safety, lack of compliance, or withdrawal of informed consent, a participant will instantly be excluded from the study. Furthermore, participants will be withdrawn from the study under the following conditions: 1) the participant’s parents require withdrawal; and 2) the participant develops heart failure, respiratory failure, or other serious disease. The DSMB might terminate the study under the following conditions: 1) SAEs occurred during the trial; 2), the investigator determined that the trial should be terminated; and 3) the Ethics Committee require termination.

#### Handling of missing data

All variables included in the CRF are mandatory. The method of last-visit-carried-forward will be used to handle the missing data.

#### Data auditing

This trial will be audited by the Research Ethics Committee and the China Food and Drug Administration. The audits will be performed when the first participant enrolls, and when half and all of the enrolled cases are completed.

### Statistical considerations

#### Sample size

The sample size was calculated based on the primary outcome measure according to the results of our pilot study. Power was set at 80% and calculated based on two-sided 95% confidence intervals. In our pilot data, we detected a post-treatment difference in PD symptoms of at least 20% between the VFS and CFS groups for SWS scores. We assume that the placebo effect can account for about 10% of such differences. We assume that 10% of the trial participants will be lost to attrition. Thus, 53 participants need to be allocated to each treatment group to establish a difference among the treatments at a level of 5% with a power value of 90%.

#### Data analysis

All evaluations of effectiveness and safety will be conducted according to the intention-to-treat principle. The final data will be analyzed using IBM SPSS 13.0 or higher (SPSS, Inc., Chicago, IL, USA) and SAS 9.4 or higher (SAS Institute Inc., Cary, NC, USA). A *p* value <0.05 will be considered statistically significant. Data will be analyzed with *t* tests and χ^2^ tests for continuous variables and categorical variables, respectively.

### Confidentiality

When participating in research there is always a risk regarding the confidentiality of information. All information gathered in the present study will be stored in a secure database. All participants will provide written informed consent prior to being assessed for eligibility for inclusion in any part of the study. Every precaution will be taken to respect the privacy of participants in the conduct of the study. Information will be stored on a password-protected server with access that is limited to members of the study team. In the course of monitoring data quality and adherence to the study protocol, the monitors will refer to medical records at the participating hospitals. All individual and site information will be de-identified when reporting the data and results to protect the confidentiality of the participants.

## Discussion

Axial and appendicular motor symptoms severely affect the quality of life of patients with PD. DBS is an effective and well-established therapy for medication-refractory patients with PD. High-frequency DBS is highly efficacious in ameliorating appendicular symptoms in patients with PD, but is less effective in improving axial symptoms, especially on a long-term basis [5]. New conceptions of DBS have focused on the use of LFS or combined STN/substantia nigra pars reticulata DBS for the treatment of axial symptoms in patients with PD [6, 9, 10]. LFS may help improve postural control as well as gait, particularly in patients with PD who do not develop gait-related disorders after HFS [11]. However, the beneficial effects of LFS on axial symptoms remain controversial [12]. Therefore, the development of novel approaches to manage both axial and appendicular motor symptoms in patients with PD is critical. Based on these findings and our previously published case report [8], we hypothesize that patients with PD and FOG might benefit from STN-VFS. This multicenter, double-blind, randomized clinical trial will enable us to evaluate the effect of a novel STN-VFS stimulation pattern on both axial and segmental symptoms of PD. This study was designed to evaluate the short-term effects and potential side-effects of VFS and CFS on axial symptoms in advanced PD. We hope to gain insight regarding whether VFS or CFS is more effective for treating axial symptoms of PD. This study has several strengths. First, group assignment in the double-blinded phase was randomized. Second, we performed a power calculation for this study. Most important of all, this new paradigm possibly offers an approach that could optimize DBS programming and improve PD DBS outcomes. At present, the total number of patients enrolled in the study is 50.

### Trial status

The trial is ongoing and is actively enrolling. The protocol version is 1.1, PINS-VFS-1601, 10 November 2016. The trial will be ongoing from August 2017 to December 2019.

## Supplementary information

**Additional file 1.** SPIRIT 2013 checklist: recommended items to address in a clinical trial protocol and related documents.

## Data Availability

Data from this randomized controlled study are unavailable at the time of publication. Individual participant data are available upon request.

## Abbreviations

AE: Adverse event
CFS: Constant frequency stimulation
CRF: Case report form
DBS: Deep-brain stimulation
DSMB: Data and Safety Monitoring Board
FOG: Freezing of gait
HFS: High-frequency stimulation
LFS: Low-frequency stimulation
PD: Parkinson’s disease
SAE: Serious adverse event
SPIRIT: Standard Protocol Items: Recommendations for Interventional Trials
STN: Subthalamic nucleus
SWS: Stand–walk–sit
UPDRS: Unified Parkinson’s Disease Rating Scale
VFS: Variable-frequency stimulation
